# Kinsenoside screening with a microfluidic chip attenuates gouty arthritis through inactivating NF-*κ*B signaling in macrophages and protecting endothelial cells

**DOI:** 10.1038/cddis.2016.255

**Published:** 2016-09-01

**Authors:** Qiao Han, Wang Bing, Yin Di, Li Hua, Li Shi-he, Zheng Yu-hua, Han Xiu-guo, Wang Yu-gang, Fan Qi-ming, Yang Shih-mo, Tang Ting-ting

**Affiliations:** 1 Shanghai Key Laboratory of Orthopaedic Implants, Department of Orthopaedic Surgery, Shanghai Ninth People's Hospital, Shanghai Jiao Tong University School of Medicine, Shanghai, People's Republic of China; 2 Department of Orthopaedic Surgery, The First Affiliated Hospital of Kunming Medical University, Kunming, People's Republic of China; 3 Complex and Intelligent Research Center, School of Mechanical and Power Engineering, East China University of Science and Technology, Shanghai, People's Republic of China; 4 Wenshan Zhengbao Orthopaedic Hospital of Yunnan Province, Wenshan, People's Republic of China

## Abstract

Gouty arthritis is a rheumatic disease that is characterized by the deposition of monosodium urate (MSU) in synovial joints cause by the increased serum hyperuricemia. This study used a three-dimensional (3D) flowing microfluidic chip to screen the effective candidate against MSU-stimulated human umbilical vein endothelial cell (HUVEC) damage, and found kinsenoside (Kin) to be the leading active component of *Anoectochilus roxburghi*, one of the Chinese medicinal plant widely used in the treatment of gouty arthritis clinically. Cell viability and apoptosis of HUVECs were evaluated, indicating that direct Kin stimulation and conditioned medium (CM) from Kin-treated macrophages both negatively modulated with MSU crystals. Additionally, Kin was capable of attenuating MSU-induced activation of nuclear factor-*κ*B/mitogen-activated protein kinase (NF-*κ*B/MAPK) signaling, targeting I*κ*B kinase-*α* (IKK*α*) and IKK*β* kinases of macrophages and influencing the expressions of NF-*κ*B downstream cytokines and subsequent HUVEC bioactivity. Inflammasome NLR pyrin domain-containing 3 (NALP3) and toll-like receptor 2 (TLR2) were also inhibited after Kin treatment. Also, Kin downregulated CD14-mediated MSU crystals uptake in macrophages. *In vivo* study with MSU-injected ankle joints further revealed the significant suppression of inflammatory infiltration and endothelia impairment coupled with alleviation of ankle swelling and nociceptive response via Kin treatments. Taken together, these data implicated that Kin was the most effective candidate from *Anoectochilus roxburghi* to treat gouty arthritis clinically.

Gouty arthritis is characterized by increased hyperuricemia level, which is triggered by disorders of the purine catabolic pathway, and leads to the deposition of monosodium urate (MSU) crystals within synovial joints and tissues.^1^ This process of an acute inflammatory attack with intense pain, swelling and skin reddening is highlighted by the influx of neutrophils into articular or periarticular tissues and the release of inflammatory cytokines after the phagocytosis of deposited MSU microcrystals and membranolysis by monocyte–macrophages,^2^ which further magnifies the inflammation cascade. The resultant stimulation of inflammatory cytokines is a cardiovascular risk factor,^3^ which contributes to the significant damage against human umbilical vein endothelial cells (HUVECs) via an increased apoptosis rate^4^ and thrombus formation.^5^ Colchicine and non-steroidal anti-inflammatory drugs (NSAIDs) (e.g., indomethacin, Indo), which are first-line treatments that inhibit this process clinically, are frequently used against acute gouty arthritis. Nonetheless, the inevitable side effects involving gastrointestinal bleeding, gastrointestinal toxicity and renal toxicity of such pharmacological reagents^6^ restrict their further utilization.^6^ Therefore, active extracts from natural herbal plants are gradually catching the attention of researchers because of the distinct merits of extensive sources, desirable efficacy and reduced adverse effects.^7^

*Anoectochilus roxburghii*, a widely used remedy for gouty arthritis in Traditional Chinese Medicine clinically, is supposed to show extensive pharmacologic effects against liver disease,^8^ hyperglycemia,^9^ hyperlipidemia^10^ and cancers.^11, 12^ So far, researchers have unraveled five major categories that constitute the active component of *A. roxburghii*, which are flavonoids, phenols, steroids, organic acids and glycosides.^13^ Despite the widely application in antigout treatment clinically, the primary functioning element of *A. roxburghii* and the underlying mechanisms still remain elusive. Furthermore, recent pharmaceutical studies of gouty arthritis primarily focused on two-dimensional static testing *in vitro* and ignored the real three-dimensional circulated circumstances that endothelial cells underwent *in vivo*. These researches highlighted the direct influence of certain drugs on inflammation or endothelial cells and neglected the intimate crosslink between inflammation and vascular complaints.^2, 5, 6, 14, 15^ Therefore, a microfluidic device, in which functionalized cells are grown on a three-dimensional flowing system instead of two-dimensional static culturing dishes, emerged to identify the major nutraceutical of *Anoectochilus roxburghii*. This device exhibits the following advantages: high throughput, sufficient flexibility^16^ and accurate modeling of an *in vivo-like* coculture context of cell–cell and cell–ECM *in vitro*,^17^ providing a promising toolkit for effective drug screening, especially for traditional medical plants.

In summary, the present study identified the major antigout candidate isolated from the widely used *A. roxburghii* under three-dimensional (3D) microfluidic conditions. The underlying protective mechanisms of this agent on MSU-induced endothelial cell injury should be investigated.

## Results

### Determination of drug concentrations in chips

**Figure 1:**
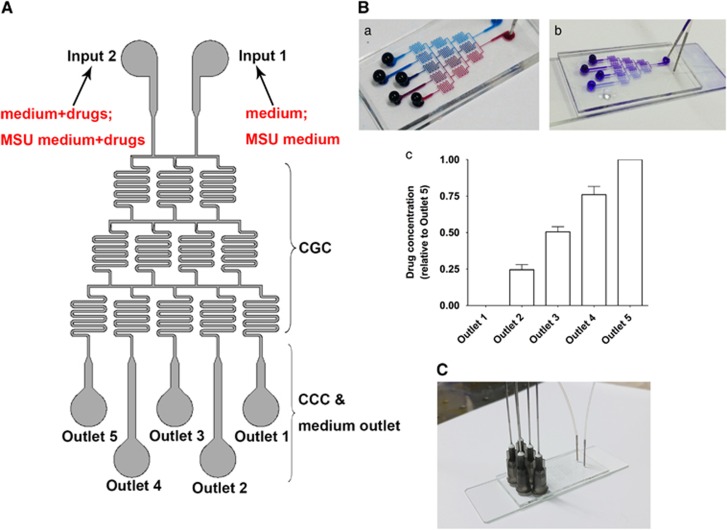
Schematic graph of the integrated microfluidic 3D flowing GCC for screening. (**A**) The *vivo-like* system consists of medium inputs, parallel CGCs and CCCs. (**B**a) Mixture of blue colorant with red colorant in CGC zone displayed a well-proportioned concentration distribution. (b) Mixture of crystal violet and PBS demonstrated gradient color depth, which reflect varying drug concentrations. (c) The indicated distribution of drug concentrations in the CCC zone. (**C**) The complete view of GCCs. The data are presented as the means±S.D. All data were obtained from at least three independent experiments

Figure 1A shows that the entire microfluidic chip (gradient culture chip, GCC) primarily contained medium inputs, parallel microfluidic channels (concentration generator channel, CGCs) and cell-culturing chambers (CCCs) to evaluate cell viability in three-dimensional flowing conditions under the stimulations of five representative components derived from *A. roxburghii*: gastrodin (Gas), kaempferol (Kae), stigmasterol (Sti), ursolic acid (Ua), kinsenoside (Kin). Figure 1Ba illustrates that the design of the GCC enabled a desirable mixture of media from two separate inputs with a gradient color background from red to blue in the CGCs. Crystal violet and PBS were used to evaluate disparate concentrations in the five CCC/outlets (Figure 1Bb). Figures 1B and C shows that the color shades gradually change from purple to transparent, and outlets 1–4 accounted for ~0%, 25%, 50% and 75% of the transformed gray scale in outlet 5, respectively, which indicates that the concentrations in outlets 1–4 also reached 0%, 25%, 50% and 75%, respectively, of the drug concentration in outlet 5. The complete view of microfluidic chip was shown in Figure 1C. Therefore, we pumped medium containing 100 *μ*M of the five candidate extracts from Input 2 and no-drug medium from Input 1, which contributed to the 0, 25, 50, 75 and 100 *μ*M in outlets 1–5, respectively.

### Microfluidic screening of effective candidate from *A. roxburghii*

**Figure 2:**
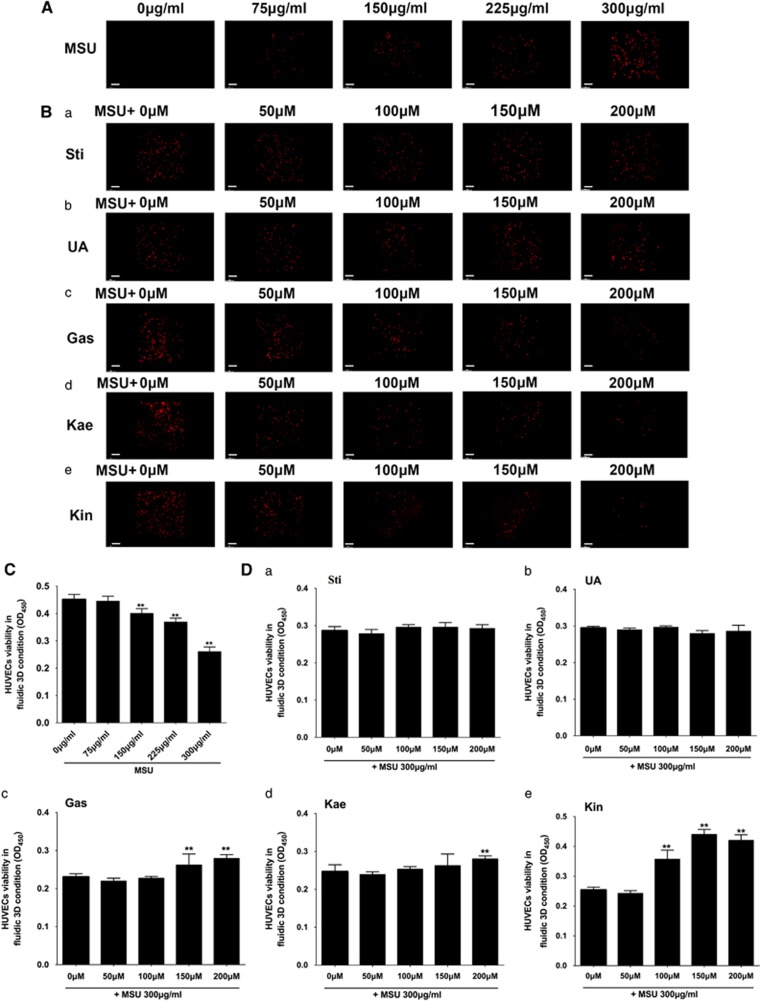
Microfluidic screening of effective candidate from *A. roxburghi.* (**A**) The apoptotic HUVECs under 3D flowing condition in response to various concentrations of MSU crystals were compared (magnification, × 100). (**B**) The apoptotic HUVECs under 3D flowing condition pre-treated with 300 *μ*g/ml MSU crystals and stimulated with varying concentrations of Sti (a), Ua (b), Gas (c), Kae (d) and Kin (e) were prepared (magnification, × 100). (**C**) The quantitative cell viability of HUVECs under 3D flowing conditions in response to various concentrations of MSU crystals were compared. (**d**) The quantitative cell viability of HUVECs under 3D flowing conditions pre-treated with 300 *μ*g/ml MSU crystals and stimulated with varying concentrations of Sti (a), Ua (b), Gas (c), Kae (d) and Kin (e) were prepared. The data are presented as the means±S.D. **P<0.05 compared with control. All data were obtained from at least three independent experiments

High concentrations of MSU crystals (150, 225 and 300 *μ*g/ml) were able to induce significant dose-dependent damage against HUVECs (Figures 2A and C). Therefore, 300 *μ*g/ml of MSU was further deployed to investigate the drugs protective efficacy against MSU crystals. Figures 2Ba, b and Da, b illustrated that Sti and Ua failed to exhibit protective efficacy against MSU-induced impairment in 3D HUVECs. However, high concentrations of Gas, Kae and Kin attenuated MSU stimulation of HUVECs (Gas: 150 and 200; Kae: 200; Kin: 100, 150 and 200 μM) (Figures 2Bc, d, e and Dc, d, e). The EC_50_ (concentrations for 50% of the maximal effect) values (concentrations for 50% of the maximal effect) of Gas, Kae and Kin were 153.6, 159.1 and 98.6 *μ*M, respectively; hence, Kin was proposed to be the most efficacious component from *A. roxburghii* against MSU-induced HUVEC damage.

### Kin protects HUVECs from MSU crystals

**Figure 3:**
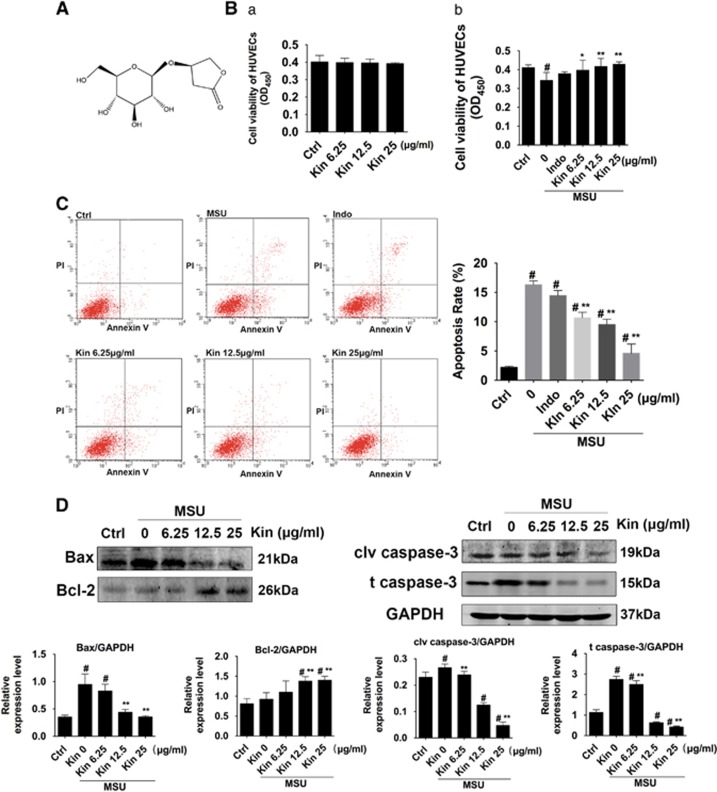
Effects of Kin on MSU-stimulated HUVECs. (**A**) The chemical structural formula of Kin. (**B**a) Cell viability were measured in Kin-treated HUVECs. (b) Cell viability were measured in HUVECs pre-treated with MSU crystals and stimulated with various concentrations of Kin. (**C**) Apoptosis rates were analyzed with HUVECs pre-treated with MSU crystals and stimulated with various concentrations of Kin. (**D**) Expressions of apoptosis-related proteins (Bax, Bcl-2, cleaved caspase-3, caspase-3) were compared in HUVECs pre-treated with MSU crystals and stimulated with various concentrations of Kin. The data are presented as the means±S.D. **P<0.05 compared with 0 *μ*g/ml of Kin. ^#^*P*<0.05 compared with control. All data were obtained from at least three independent experiments

To determine the effects of Kin on MSU-stimulated HUVECs, further experiments were performed in 96-well plates. Kin did not exhibit cytotoxicity against HUVECs at concentrations from 6.25 *μ*g/ml (25 *μ*M) to 25 *μ*g/ml (100 *μ*M) (Figure 3Ba). When HUVECs were exposed to 300 *μ*g/ml of MSU, cell viability was inhibited significantly (Figure 3Bb). Nonetheless, despite that Indo (20 *μ*g/ml) failed to enhance HUVEC proliferation after MSU intervention, Kin exhibited protective effects on MSU-stimulated HUVECs with increased viability compared with MSU treatment alone. Flow cytometry (Figure 3C) further demonstrated that Kin treatments (6.25–25 *μ*g/ml) exerted beneficial effects on MSU-augmented cell apoptosis compared with the Indo group 14.46±0.62%, as exemplified by the 10.67±0.66%, 9.52±0.64% and 4.61±1.12% of apoptosis rates from 6.25, 12.5 to 25 *μ*g/ml in the Kin groups, compared with 16.32±0.46% in the MSU group and 2.21±0.13% in the control group. These results were also confirmed using western blotting (Figure 3D), with the expressions of Bax, caspase-3, cleaved caspase-3 (proapoptotic proteins) increased and Bcl-2 (antiapoptotic protein) decreased in a dose-dependent manner in the MSU group, signifying that the enhancement on apoptosis was achieved via MSU stimulation. Besides, various treatments of Kin decreased the expressions of Bax and cleaved caspase-3 and increased the level of Bcl-2, implicating the potential antiapoptosis effects of Kin on MSU-induced HUVECs.

### Kin prevents the impairment of HUVECs from MSU-CM of macrophage cells

**Figure 4:**
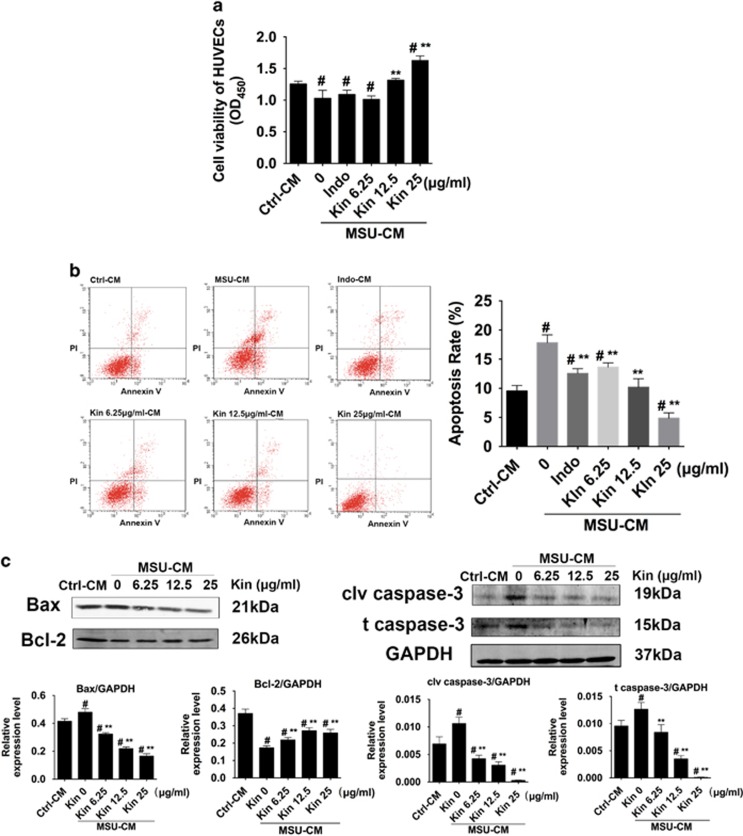
Effects of Kin-treated MSU-CM from macrophages on HUVECs. (**a**) Cell viability were measured in HUVECs treated with CM from both MSU crystals and Kin-treated macrophage cells. (**b**) Apoptosis rates were analyzed with HUVECs treated with CM from both MSU crystals and Kin-treated macrophages. (**c**) Expressions of apoptosis-related proteins (Bax, Bcl-2, cleaved caspase-3, caspase-3) were compared in HUVECs treated with CM from MSU crystals and Kin-treated macrophages. The data are presented as the means±S.D. **P<0.05 compared with 0 *μ*g/ml of Kin. ^#^*P<*0.05 compared with control. All data were obtained from at least three independent experiments

Figure 4a reveals that conditioned medium (CM) from merely MSU-treated macrophage cells was able to inhibit the growth of HUVECs. Nonetheless, when RAW264.7 cells were administered with various concentrations of drugs (Indo 20; Kin 6.25–25 *μ*g/ml) and MSU, the CMs collected were considered to enhance cell proliferative abilities of HUVECs at 12.5 and 25 *μ*g/ml of Kin even though low concentration of Kin. Indo failed to demonstrate protective properties. Annexin V/PI double staining revealed that high concentrations of Kin (12.5 and 25 *μ*g/ml) decreased MSU-CM-induced apoptosis of HUVECs. However, a low concentration of Kin (6.25 *μ*g/ml) and Indo (20 *μ*g/ml) exhibited antiapoptotic capabilities despite the negligible protective effects on cell growth (Figure 4b) (MSU: 17.82±0.96% Indo: 12.55±0.57% 6.25 *μ*g/ml Kin: 13.63±0.52% 12.5 *μ*g/ml Kin: 10.18±1.03% 25 *μ*g/ml Kin: 4.86±0.65%). Additionally, the expressions of Bax, cleaved caspase-3 and caspase-3 decreased and Bcl-2 increased compared with MSU-CM-treated group when HUVECs were added with both Kin- and MSU-treated CM from RAW264.7 cells, which suggests that the CM from inflammatory macrophages had a vital role in exhibiting protective properties against HUVECs (Figure 4c). Thus, we proposed that the efficacious regulative mechanisms of Kin might be ascribed to the adjusted CM from macrophages and thereby affecting HUVECs and the subsequent evolution of gouty arthritis.

### Kin inhibits the MSU-induced activation of NF-*κ*B and JNK/Erk

**Figure 5:**
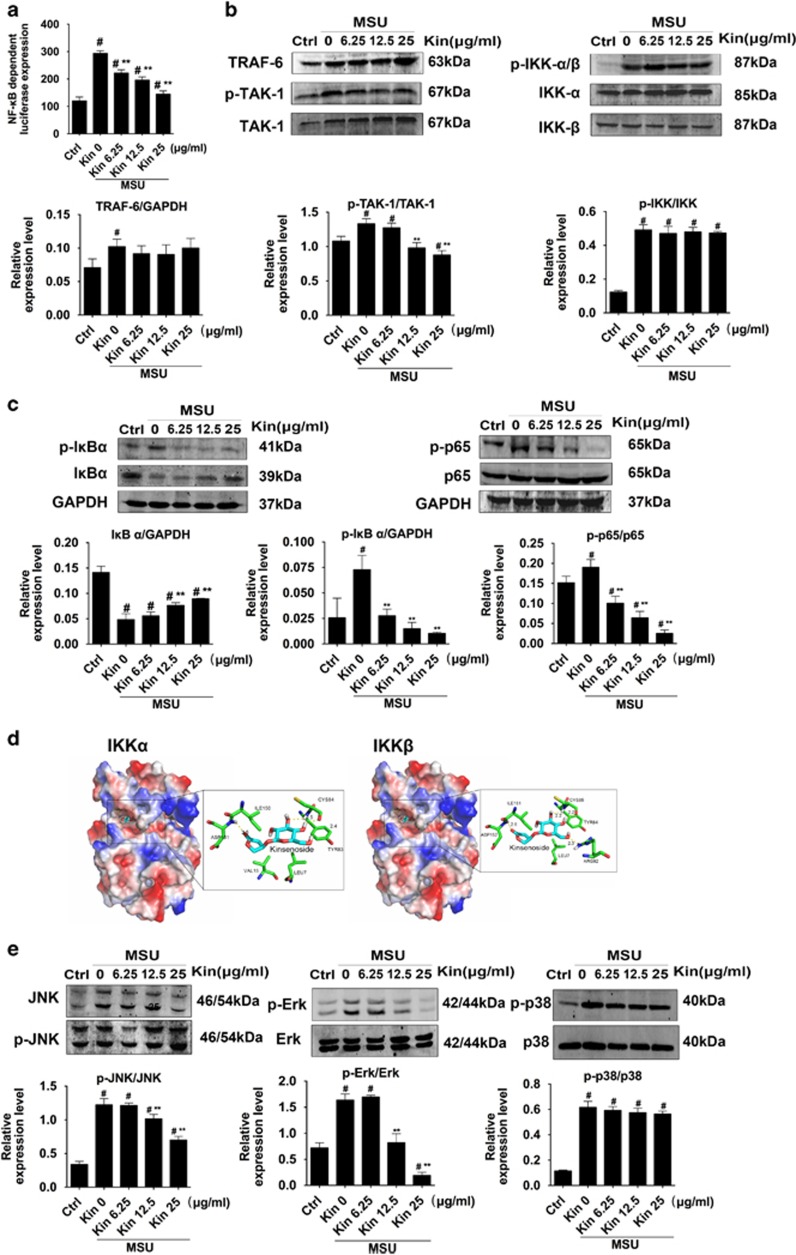
Effects of Kin on MSU-induced NF-*κ*B/MAPK activation. (**a**) Transfected RAW264.7 macrophage cells were incubated with Kin and MSU for 7 h. The luciferase activity for NF-*κ*B was evaluated and normalized to the control. (**b**) Expression of NF-*κ*B signaling-associated upstream proteins (TRAF-6, p-TAK-1/TAK-1, p-IKK*αβ*/IKK*αβ*) were compared in macrophages treated with MSU crystals and Kin. (**c**) Expression of NF-*κ*B signaling-associated downstream proteins (p-I*κ*B*α*/I*κ*B*α*, p-p65/p65) was compared in macrophages treated with MSU crystals and Kin. (**d**) Molecular docking of IKK*α* and IKK*β* with Kin. (**e**) Expressions of MAPK signaling-associated proteins (p-JNK/JNK, p-Erk/Erk, p38/p-p38) were compared in macrophages treated with MSU crystals and Kin. The data are presented as the means±S.D. **P<0.05 compared with 0 *μ*g/ml of Kin. ^#^*P<*0.05 compared with control. All data were obtained from at least three independent experiments

Nuclear factor-*κ*B (NF-*κ*B) luciferase reporter gene activity assay (Figure 5a) indicated that NF-*κ*B transcription increased significantly following MSU stimulation. Kin administrations decreased NF-*κ*B activation dose-dependently. Moreover, treatment with Kin barely affected the MSU-stimulated upstream TRAF-6, p-TAK-1 and p-IKK proteins expressions (Figure 5b) while decreasing the expressions of p-I*κ*B*α* plus p-p65 and increasing the production of inhibitor of NF-*κ*B (I*κ*B*α*) (Figure 5c), implying that Kin probably targeted IKK-*α* and IKK-*β* kinases, thus inhibiting downstream activation of NF-*κ*B. These results were further confirmed using a molecular docking assay, which demonstrated that Kin bound to ASP151, CYS84 and TYR83 in IKK-*α* and ILE151, ARG92, TYR84 and CYS85 in IKK-*β* (Figure 5d), providing potential inhibition sites that Kin functioned against the NF-*κ*B pathway. Similarly, administration of MSU increased the expression of p-JNK (c-Jun N-terminal kinase), p-Erk (extracellular signal-regulated kinase) and p-p38 in macrophages. Nonetheless, Kin was able to attenuate the expression of MSU-induced activation of p-JNK and p-Erk dose-dependently irrespective of p-p38 (Figure 5e).

### Inhibition of NF-*κ*B downstream inflammatory cytokine expressions and inflammasomes

**Figure 6:**
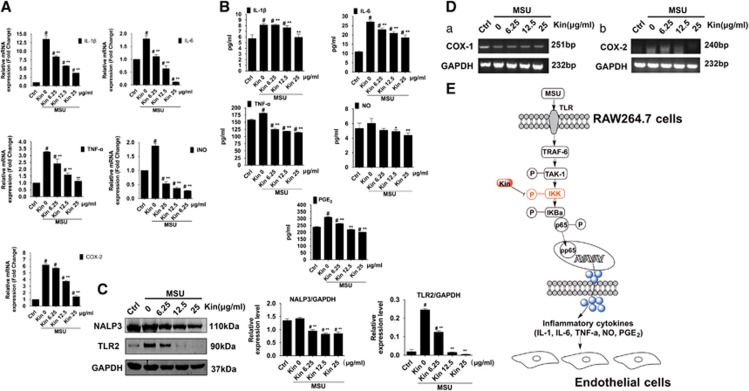
Effects of Kin on the expression of MSU-induced NF-*κ*B downstream inflammatory cytokines and inflammasome and schematic diagram of the proposed mechanisms of antigouty arthritis through modulation of macrophages. (**A**) mRNA expressions of NF-*κ*B downstream IL-1*β*, IL-6, TNF-*α*, NO and PGE_2_ in macrophages treated with MSU crystals and Kin. (**B**) Measurement of the secretion of NF-*κ*B downstream IL-1*β*, IL-6, TNF-*α*, NO and PGE_2_ in macrophages treated with MSU crystals and Kin. (**C**) Expressions of NALP3 and TLR2 were compared in macrophages treated with MSU crystals and Kin. (**D**) Effects of Kin on MSU-stimulated expression of COX-1 in HUVECs and COX-2 in inflammatory macrophages. (**E**) Kin inactivates NF-*κ*B pathway via targeting of IKK kinases, abrogating phosphorylation of I*κ*B*α* and p65, downregulating expression of IL-1*β*, IL-6, TNF-*α*, NO and PGE_2_ in macrophages, attenuating MSU-mediated HUVECs impairment. The data are presented as the means±S.D. **P<0.05 compared with 0 *μ*g/ml of Kin. ^#^*P<*0.05 compared with control. All data were obtained from at least three independent experiments

As shown in Figure 6a, the mRNA expressions of NF-*κ*B-related inflammatory cytokine genes (*IL-1β*, *IL-6*, *TNF-α*, *iNO* and *COX-2*) were significantly promoted with MSU stimulation. Nonetheless, the inductions of these genes were repressed markedly by the addition of Kin in a dose-dependent manner. Enzyme-linked immunosorbent assay (ELISA) (Figure 6b) illustrated that expressions of IL-1*β*, IL-6, TNF-*α*, NO and prostaglandin E_2_ (PGE_2_) in CM from macrophage cells were enhanced owing to MSU stimulation, with a dose-dependent decrease in MSU-induced inflammatory cytokines from macrophages after Kin treatment.

The effects on inflammasome of macrophages after Kin administration were also evaluated. We found that Kin treatments inhibited the NLR pyrin domain-containing 3 (NALP3) expression in MSU-stimulated macrophages. Also, TLR2 expression was significantly enhanced after MSU administration and various Kin treatments attenuated MSU-stimulated TLR2 expressions dose-dependently (Figure 6c).

### Selective inhibition of MSU-induced COX-2 in macrophages

We also assessed the MSU-induced expression of cyclooxygenase-1 (COX-1) in HUVECs and cyclooxygenase-2 (COX-2) in inflammatory macrophages. MSU intervention decreased COX-1 in normal endothelial cells and increased COX-2 in inflammatory cells, indicating the normal physiologic behavior of HUVECs was damaged via COX-1 decrease and the inflammatory response of macrophages was induced via COX-2 increase. Kin administration barely influenced the inhibition of COX-1 expression, but it markedly and dose-dependently diminished the MSU-stimulated expression of COX-2 in macrophages (Figure 6d), which suggested that Kin could serve as a novel promising selective COX-2 inhibitor.

### Attenuated MSU uptake in macrophages after Kin treatment

**Figure 7:**
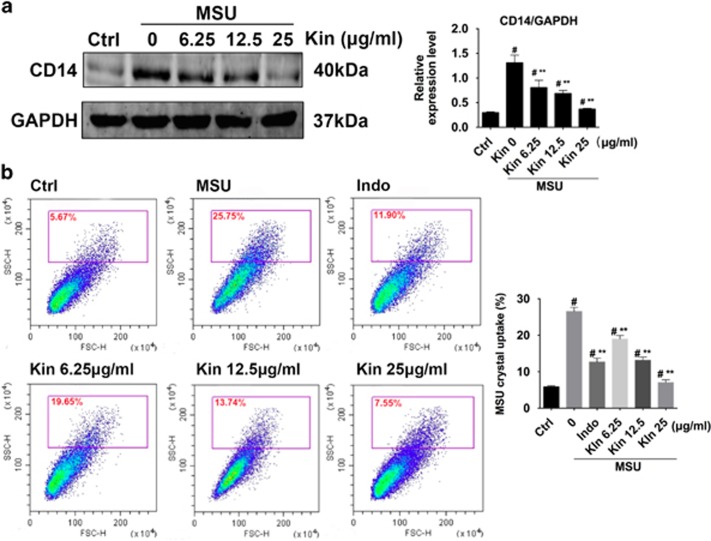
Determination of CD14-mediated MSU crystals uptake in macrophages after Kin. (**a**) Expressions of CD14 were compared in macrophages treated with MSU crystals and Kin. (**b**) Uptake of MSU crystals was analyzed with flow cytometry as an increase in the macrophage side-scatter high (SSC-H). The data are presented as the means±S.D. **P<0.05 compared with 0 *μ*g/ml of Kin. ^#^*P*<0.05 compared with control. All data were obtained from at least three independent experiments

MSU enhanced CD14 expression in macrophages, whereas Kin abrogated the MSU-induced production of CD14 dose-dependently (Figure 7a). Consistently, macrophages with MSU exhibited higher crystal uptake (side-scatter high^18^) compared with control macrophages. Kin administrations inhibited MSU uptake in macrophages dose-dependently (Figure 7b). Altogether, Kin inhibited MSU-induced CD14 expression on macrophages and therefore attenuated following phagocytosis activity.

### Histological evidence of Kin against MSU-induced gouty arthritis in rats

**Figure 8:**
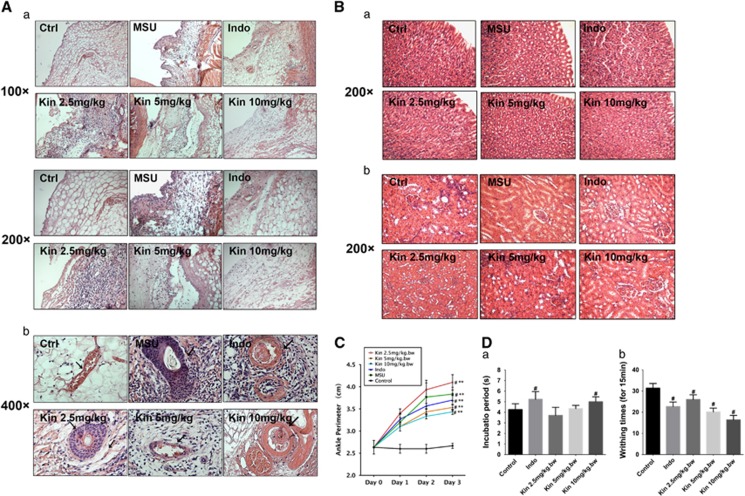
*In vivo* examination of anti-inflammatory effects of Kin against gouty arthritis. (**A**) Histological analyses of rat ankle joints injected with MSU crystals and corresponding drugs (a, magnification, × 100, × 200; b, magnification, × 400). Black arrows indicate MSU-affected blood vessels. (**B**) Histological analyses of stomach (a) and kidney (b) after rats received injection of MSU crystals and corresponding drugs (magnification, × 200). (**C**) Perimeter assessments of ankles of rats injected with MSU crystals and corresponding drugs. (**D**) The effects of Kin on nociceptive reactions caused by hot water (tail-flick response, a) and acetic acid (writhing response, b). The data are presented as the means±S.D. **P<0.05 compared with 0 *μ*g/ml of Kin. ^#^*P*<0.05 compared with control. All data were obtained from at least three independent experiments

Figure 8Aa illustrated that the infiltration of synovial membranes was significantly escalated in rats treated with MSU crystals compared with PBS-treated rats. However, the MSU-induced inflammatory penetration decreased remarkably following the administration of Indo and Kin (5 and 10 mg/kg), but 2.5 mg/kg Kin exhibited no obvious anti-inflammatory effects. Additionally, the protective efficacy of endothelial cell exerted by Kin against MSU crystals *in vivo* was emphasized. Figure 8Ab shows that without MSU stimulation, we observed no obvious macrophagocytes migration into vascular ring muscle, as well as the relatively thin interlayer of vessel. Nonetheless, a large number of neutrophils migrated into the vascular ring muscle in the synovial membrane of MSU-treated rats, which contributed to the thicker interlayer of blood vessels. In contrast, no obvious evidence of inflammatory leukocyte penetration in Indo-treated gouty arthritis rats, which exhibited thinner vessel walls, was observed. Furthermore, Kin administration decreased the thickness of the vessel ring muscle dose-dependently with diminished inflammatory infiltration into the medial layer of the vessel wall of neutrophils. Particularly, the endothelial-protective effect of high-dose Kin (10 mg/kg) was also superior to Indo treatment, which indicates its potential as a promising pharmaceutical reagent against gouty arthritis. Additionally, as shown in Figure 8Ba, b, the gastric gland arrangement and renal structure remained intact after Kin treatments.

### Attenuation of MSU-induced ankle swelling, tail-flick response and writhing reaction

The ankle perimeter of urate-induced right hindlimbs increased significantly within 3 days compared with control rats. Indo (5 mg/kg) plus 10 mg/kg Kin administration markedly reduced the MSU-induced ankle swelling compared with the model group, but 2.5 mg/kg Kin barely exerted an antiarthritic effect (Figure 8C). Figure 8Da shows that the Indo group and the 10 mg/kg Kin group significantly increased the incubation period of the tail-flick response compared with the control group and increased the pain threshold 42.08±14.44% and 28.02±4.43%, respectively, irrespective of 2.5 and 5 mg/kg of Kin-treated rats. Indo and Kin treatments (2.5, 5 and 10 mg/kg) significantly decreased the cumulative number of acetic acid-induced writhing responses. The analgesic rates were 28.8±5.6%, 18.5±4.4%, 35.3±4.7% and 48.5±2.9%, respectively, relative to control rats (Figure 8Db).

## Discussion

In this study, Kin was used to treat MSU-stimulated HUVECs based on a flowing three-dimensional microfluidic system that was deployed to screen the leading active candidate from five representative elements of *Anoectochilus roxburghii*. Kin has been proven to suppress LPS-stimulated inflammatory response in mouse peritoneal lavage macrophages,^19^ inhibiting RANKL-stimulated osteoclastogenesis via NF-*κ*B pathway^20^ and decreasing blood glucose levels in diabetic mice, indicating a promising potential for the treatment of osteoporosis and diabetic vascular diseases.^21^ Moreover, the administration of Kin significantly repressed inflammatory IL-1*β*, IL-17, IFN-*γ*, TNF-*α* and MMP-9 cytokines production and bone destruction in mice with collagen-induced rheumatoid arthritis.^22^ However, no existing study shows the antigouty arthritis effects of Kin through modulation of macrophages. Herein, we pioneered to unravel that Kin was capable of targeting IKK kinases in macrophages, thus inhibiting the activation of NF-*κ*B signaling and ameliorating MSU-induced HUVECs impairment.

Endothelial cells inhabit in 3D architectures in host microenvironments. Nonetheless, currently, the majority of *in vitro* drug-screening researches were conducted in two-dimensional culturing systems.^23, 24, 25^ The genotypic and phenotypic bioactivity of stromal cells in 2D system may diverge tremendously from authentic 3D state owing to the absence of essential modulation of extracellular microenvironments.^26, 27^ The 3D systems vividly model the interior coculture context, by which flowing environments can be recreated for antigouty arthritis research. So far, many studies have shown that microfluidic system was able to not only simulate the shear stress, interstitial and blood flow that endothelial network exposed to^28^ but also assay the efficacy of anticancer drug sensitivity under such *vivo-resemble* microenvironment.^29^ Therefore, a 3D culturing system that HUVECs endured was constructed with consecutively flowing drug stimulation, providing authentic screening results from *Anoectochilus roxburghii*. We discovered that Kin screening from microfluidic chips was more effective than other representative candidates against MSU-induced impairments of endothelial cells with the lowest value of EC_50_ in 3D *vivo*-like condition. Further results confirmed that non-toxic Kin doses also prevented MSU-intervened proliferation and apoptosis of HUVECs directly. MSU hass an important role during acute arthritis and vascular disorder of joint, which represents a well-established prognosis marker in gouty arthritis patients with obesity, hypertension, diabetes and chronic kidney disease.^30^ MSU crystal deposition in synovial tissues produces an influx and phagocytosis of monocyte–macrophages, and the release of cytokines further influences the bioactivity of endothelial cells.^2, 31, 32^ Kin improved HUVECs viability and inhibited HUVECs apoptosis caused by the CM of MSU-treated macrophage cells. Various Kin concentrations directly and indirectly inhibited MSU-induced growth arrest and apoptosis of HUVECs with better efficacy compared with Indo treatment. Therefore, Kin may target inflammatory cells and safeguard the regular biological activities of endothelial cells.

As noted above, the activation of NF-*κ*B signaling serves as the central mediator of inflammation response that impresses the productions of inflammatory enzymes and cytokines.^33^ TLR2 at the plasma membrane interacts with MSU crystals,^18^ and CD14 serves as adaptor molecule that enable the recognition of MSU to TLR2 and further stimulated crystals uptake and downstream canonical signal pathway.^34^ The activation of TLR2 upon MSU binding induces an upregulation of TRAF-6 and TAK-1 phosphorylation.^35, 36^ Subsequently, the IKK complex, which is comprised of catalytic IKK*α*, IKK*β* and IKKγ subunits, phosphorylates I*κ*B*α*, which activates the phosphorylation of I*κ*B*α* and its subsequent degradation. This action is followed by the increased translocation and phosphorylation of downstream p65.^37^ Similarly, the activation and phosphorylation of MAPKs (p-JNK/JNK, p-Erk/Erk and p-p38/p38) enact crucial roles during various cell behaviors, involving proliferation, apoptosis and inflammation response.^38, 39^ We found that the transcription of NF-*κ*B increased markedly in the presence of MSU, and the addition of Kin dose-dependently attenuated NF-*κ*B activation. Kin remarkably inhibited the degradation of I*κ*B*α* and phosphorylation of I*κ*B*α* and downstream p65 without influencing the upstream IKK subunits. These results suggested that Kin bound IKK kinases and inhibited corresponding function, which affected the downstream I*κ*B*α*/p-I*κ*B*α*. Computational analyses of molecular docking indicated that Kin bound IKK*α* and IKK*β* at specific docking sites, which confirmed our hypothesis that IKKs are targets for Kin. Additionally, the activation of MSU-activated MAPK pathway was repressed with Kin treatment significantly, which might contribute to the regulative inflammation mechanisms upon HUVECs partially.

The expression of IL-1*β*, IL-6, TNF-*α*, NO and PGE_2_ is mediated via NF-*κ*B signaling.^40, 41, 42^ NALP3 inflammasome, a vital innate immune pathway that modulates the secretion of inflammatory cytokines, is activated by a variety of bacterial toxins and crystals.^43, 44^ We found that Kin treatment dose-dependently attenuated IL-1*β*, IL-6, TNF-*α*, NO and PGE_2_ via suppression of NF-*κ*B, which diminished HUVEC damage from Kin-treated macrophages.^14, 31^ Also, Kin inhibited MSU-induced expressions of NALP3 and TLR2, which contributed to the attenuated inflammation in gouty disease.

NSAIDs are ubiquitously prescribed in clinics to provide anti-inflammatory and analgesic properties via COX inhibition in gout arthritis treatments.^45^ COX that supposes to determine the production of prostanoid from arachidonic acid (AA) comprises two isoforms: COX-1, the housekeeping enzyme that is responsible for hemostatic integrity and gastric cytoprotection, and COX-2, the inducible enzyme that reacts to external stimuli and promotes the inflammatory reaction.^46, 47^ The severe side effects such as gastric bleeding, mucosa erosion and ulcers are unavoidable of NSAIDs such as Indo, which are primarily the results of suppression of the COX-1-derived protective PGE_2_. Kin dose-dependently abrogated MSU-induced COX-2 expression in macrophages without affecting the MSU-stimulated COX-1 decrease in HUVECs. Despite that gout patients take COX-2 inhibitor only for few days, and that the activation of COX-2 has a vital role during other inflammatory arthritis and cancer progression,^48^ the selective COX-2 inhibition of Kin could be used in many aspects besides gout arthritis such as anti-inflammation and anticancer strategies.

The influx of a vast number of mononuclear phagocytes into joint synovial tissue is the key feature of acute gouty arthritis.^49^ To evaluate more closely the role of Kin against gouty arthritis, we established a murine gouty arthritis model through intra-articular injection of MSU crystals. This procedure triggered a series of inflammatory reactions resemble to that of acute gouty arthritis.^50^ In MSU-injected ankle joints, Kin treatments alleviated inflammatory infiltration and ankle perimeter. The impairment of HUVECs produces a thicker interlayer of blood vessels with inflammatory neutrophil infiltration.^51^ Kin administration dose-dependently decreased the thickness of the vessel ring muscle with diminished inflammatory infiltration into the medial layer vessel wall of neutrophils, which was better than the efficacy of Indo *in vivo*. As for intense pain control,^52^ the tail-flick response reflects central acting analgesic effect.^53^ Results showed that high concentration of Kin extended reaction duration, whereas low concentration of Kin failed to show antinociceptive effects. The writhing response evaluates peripheral acting analgesic effect,^54^ and results illustrated that Kin attenuated acetic acid-induced intraperitoneal response, with increased analgesic rate. The antinociceptive effects of Kin were superior to Indo. Our animal experiments demonstrated an obvious suppression of MSU-induced inflammatory infiltration and endothelium damage with evident amelioration of ankle swelling and nociceptive reaction via Kin treatment *in vivo*.

Undoubtedly, the progress of cancer, microbial infection and other chronic arthritis require the active participation of macrophages. Previous researches revealed that inflammatory cells could serve as potential targets to achieve desirable efficacy during the treatments of tumor, microbial infection and inflammatory bone diseases.^55, 56, 57, 58^ Therefore, we consider that the treatment of Kin could be applied in the therapies of cancer, microbial infection and inflammatory diseases, which require future in-depth work.

In conclusion, *vivo-like* 3D flowing microfluidic chip screening *in vitro* demonstrated that Kin was the most effective candidate from *A. roxburghii* against MSU-induced gouty disease. Various Kin treatments suppressed the MSU-stimulated proliferation inhibition and apoptosis induction of HUVECs *in vitro* and *in vivo* via targeting of IKK*α* and IKK*β* kinases in macrophages, which repressed NF-*κ*B signaling and related cytokine expressions and subsequent endothelium bioactivity. Our study provides a deeper understanding of gouty arthritis and suggests that Kin is a novel COX-2-selective inhibitor for the clinical treatment of gouty arthritis.

## Materials and Methods

### Cells, media and reagents

HUVECs and murine RAW264.7 macrophage cells were cultured with Dulbecco's minimum essential medium (DMEM; HyClone, Logan, UT, USA) containing 10% fetal bovine serum (HyClone), penicillin 100 U/ml and streptomycin 100 *μ*g/ml (Gibco, Invitrogen Ltd, Carlsbad, CA, USA) at 37 °C in humidified conditions with 5% CO_2_. CM contained serum-free DMEM medium and 5% bovine serum albumin (Sigma-Aldrich, St. Louis, MO, USA). MSU crystals and Indo were purchased from Sigma-Aldrich and stored in dark at room temperature. Inclusion criterion of selected drugs (Sti, Ua, Kae, Kin and Gas) were: (1) constitute the majority of *A. roxburghii*; (2) represents each specific type of *A. roxburghii* (Gas from flavonoids, Kae from phenols, Sti from steroids, Ua from organic acids and Kin from glycosides). Sti, Ua and Gas^13^ were purchased from Sigma-Aldrich. Kae and Kin^13^ were purchased from Shifeng Biological Technology Company (Shanghai, China).

### Fabrication of microfluidic chip

The entire microfluidic chip (GCC) mainly contained medium inputs, parallel microfluidic channels (CGC) and CCC. Two Harvard injection pumps that connected to the inputs were used to manipulate the medium flow in chip. The GCC was manufactured with polydimethylsiloxane (PDMS) by repeated molding on a master, which was fabricated with a 100 mm layer of SU8-2035-negative photoresist onto a glass wafer and shaped by photolithographic methodology.^59^ PDMS base and curing agent were mixed violently (mass ratio at 10:1), degasified under vacuum and poured onto the master. After 1 h heating at 80 °C and subsequent cooling, PDMS layer was peeled carefully from the master and stick irreversibly to a glass substrate (1.2 mm thick) after biong treated with oxygen plasma for 2 min. Such fabricated microfluidic GCC chip was immersed into ddH_2_O and sterilized under ultraviolet before use.

### Determination of drug concentration distribution in chips

The determination of drug concentration in microfluidic GCC was based on the flow field distribution.^60, 61^ The CGC zone deployed zigzag design so as to mix different medium from inputs sufficiently. Crystal violet and PBS were used to establish varying drug concentrations that could be reflected by the color depth of crystal violet.

### Drug administration via microfluidic chip

HUVECs were harvested and resuspended at a concentration of 2.5 × 10^5^/ml in cell-basement membrane extract (BD Bioscience, San Jose, CA, USA) to create a 3D format.^62^ A cell-gel mixture (20 *μ*l) was seeded into each CCC before the covering of syringe needle, which allowed the effusion of the inner medium to the outer collecting pipes. 3D endothelial cells were initially treated with various concentrations of MSU crystals (0–300 *μ*g/ml) to validate the optimal urate stimulation dose. Cells pre-treated with culturing medium with and without drugs (100 *μ*M) were further stimulated with MSU medium (300 *μ*g/ml) with and without drugs (100 *μ*M), and then pumped through Input 1 and Input 2, respectively (10 *μ*l/min).

### 3D flow drug efficacy assay

Ethidium homodimer (Invitrogen, Carlsbad, CA, USA) that binds to nucleic acid of the dead cells and produce red fluorescence was used through the chip for 30 min.^63^ The apoptotic cell staining was observed under confocal laser scanning microscope (Leica, Wetzlar, Germany). For quantitative analysis, equal volume of Cell Counting Kit-8 (CCK-8) solution was pumped into the chip for 3 h through two inputs (3 *μ*l/min) and the collected CCK-8 from five outlets were measured at the optical density (OD) of 450 nm.

### 2D drug efficacy assay

HUVECs (6000 cells per well) were then seeded in a 96-well plate, pre-treated with culturing medium or CM containing Kin (6.25–25 *μ*g/ml) and Indo (20 *μ*g/ml) and stimulated with MSU (300 *μ*g/ml) for 24 h. The CCK-8 assay was performed, and the OD_450_ values were measured.^64, 65, 66^

### Flow cytometry

For Annexin V/propidium iodide (PI) immunofluorescence, HUVECs were pre-treated directly with DMEM added with drugs and MSU crystals for 24 h. Meanwhile, RAW264.7 macrophages were pre-treated with DMEM containing Kin (6.25–25 *μ*g/ml) and Indo (20 *μ*g/ml) and stimulated with MSU (300 *μ*g/ml) for 24 h. The CM supernatants of macrophages were collected to further administer with HUVECs for 24 h. Cell apoptosis levels of HUVECs from both administrations were determined with flow cytometry via Annexin V/PI staining according to the manufacturer's instructions (BD Bioscience).^67^ The percentages of apoptotic HUVECs were presented.

To measure the MSU crystal uptake by macrophages, RAW264.7 macrophages were pre-treated with DMEM containing Kin (6.25–25 *μ*g/ml) and Indo (20 *μ*g/ml) and stimulated with MSU (300 *μ*g/ml) for 24 h. Cells were then fixed in 4% paraformaldehyde and side-scatter high analysis was performed to determine the crystal uptake in macrophages according to previous report.^18, 68^

### Western blot

Macrophages were pre-treated with DMEM containing Kin (6.25–25 *μ*g/ml) and Indo (20 *μ*g/ml) and stimulated with MSU (300 *μ*g/ml). The CM supernatants of macrophages were collected for administration to HUVECs. HUVECs were pre-treated directly with culturing medium supplemented with drugs and MSU crystals. Proteins were extracted and used for western blot analyses, as reported previously.^69^

### RNA extraction, real-time PCR and reverse transcription-PCR

HUVECs and macrophage cells were pre-treated with DMEM containing Kin (6.25–25 *μ*g/ml) and stimulated with MSU (300 *μ*g/ml) for 24 h. Then, RNA was extracted using TRIzol method. The mRNA expressions of IL-1*β*, IL-6, TNF-*α*, iNO and COX-2 in macrophages and COX-1 in HUVECs were measured with real-time PCR (ABI 7500; Applied Biosystems, Foster City, CA, USA) and reverse transcription-PCR.^67^ (IL-1*β*: 5′-CCTCCAGGAAGGAGCAAAAC-3′ IL-6: 5′-GCCTTCTTGGGACTGAT-3′ TNF-*α*: 5′-ATGGATCCACCATGAGCACAGAAAGC-3′ iNO: 5′-CCCCATCAAGCCCTTTACTT-3′ COX-2: 5′-CTTACAATGCTGACTATGGCTAC-3′ COX-1: 5′-CTACAACACGGCACACGACT-3′).

### Cytokine measurements

Macrophages were pre-treated with Kin (6.25–25 *μ*g/ml) and MSU (300 *μ*g/ml) in DMEM for 24 h. The supernatants were collected and the concentrations of NO, PGE_2_, IL-1*β*, IL-6 and TNF-*α* were measured via ELISA Kits (BioSource, Camarillo, CA, USA) according to the manufacturer's guidelines.

### NF-*κ*B luciferase reporter gene assay

RAW264.7 macrophages stably transfected with a p-NF-*κ*B-TA-Luc luciferase reporter construct were used to evaluate the effects of Kin on MSU-induced NF-*κ*B activation according to a previous report.^70^ The cells were pre-treated with Kin and MSU for 7 h, and luciferase activities were determined using the Promega Luciferase Assay System (Promega, Madison, WI, USA), following the manufacturer's instructions.

### Molecular modeling

The three-dimensional homology models of mouse IKK*α*/*β* kinase domains were established with Modeler 9.12 using the architectures of human IKK*α*/*β* as templates. The stereochemical constructs of IKK*α*/*β* were confirmed using PROCHECK. MolConverter and MarvinSketch (ChemAxon, Budapest, Hungary) were used to generate and refine the spatial coordinates of the Kin drug. Kin was linked to the ATP docking pockets of IKK*α* and IKK*β* using the Lamarckian genetic algorithm based on AutoDock and AutoDock Vina.^71^ The consequential molecular modeled figures of binding activities were prepared using the PyMOL visualization software (Schrodinger LLC, New York, NY, USA).

### Animal model of MSU-induced ankle gouty arthritis

A total of 80 male Sprague–Dawley rats (200±20 g) were purchased from Shanghai Slac Laboratory Animal Company (Slac, Shanghai, China) and fed commercial food and water in specific pathogen-free conditions approved by Animal Ethical Committee of Shanghai Ninth Hospital. Thirty rats were randomly divided into six groups (five rats per cage). Group 1 rats were injected daily with 100 *μl* of 0.9% physiological saline into the ankle of their right hind legs consecutively for 3 days. For Groups 2–6, 100 *μ*l MSU crystals (10 mg/ml) was administered into the rat ankle joint of the right lower limb 1 h before an intraperitoneal injection of 500 *μl* Indo (5 mg/kg per body weight (bw)) in Group 3 and 500 *μ*l Kin (2.5, 5 and 10 mg/kg per bw) in Groups 4–6, respectively. Drugs and MSU crystals were administered daily for 3 days, and the circumferences of injected ankle joints were measured before each MSU injection.

### Histological observation

All of the animals were killed 3 days after the drug and MSU interventions, and the injected ankle joints of the right hindlimbs as well as kidney and stomach tissues were harvested and fixed in 4% paraformaldehyde. Synovial tissues from ankle joints and kidney and stomach tissues were paraffin-embedded and sectioned for hematoxylin and eosin staining.

### Behavioral testing: tail-flick response and writhing response

According to the well-established methods,^72^ 50 rats were randomly assigned into five groups (five rats per cage): control (Group 1, 0.9% physiology saline, intraperitoneally), Indo (Group 2, 5 mg/kg bw, intraperitoneally), Kin at low (Group 3, 2.5 mg/kg bw, intraperitoneally), medium (Group 4, 5 mg/kg bw, intraperitoneally) and high (Group 5, 10 mg/kg bw, intraperitoneally) concentrations (*n*=10). All animals received intra-articular injection of MSU crystals before further behavioral testing. All the required drugs were given intraperitoneally daily for consecutive 7 days. The tail-flick latency was evaluated 1 h after the last drug administrations on the seventh day. A constant hot water of 55±1 °C was retained and the distant end of rat tail (5 cm) was submerged into hot water and the latent interval between tail submergence and tail flick was recorded with a stopwatch (s). Also, on the first day before drug interventions, the basal pain threshold (s) was marked to compute the pain latency and the pain threshold increase ratio (%) with below formula:^52^


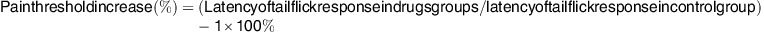


The writhing response was assessed with acetic acid (0.7%) (15 ml/kg bw) that was injected into each rat intraperitoneally 15 min after final drugs administration on the seventh day. The intensity of writhing response was measured with the accumulative number of writhing activities within duration of 15 min since acetic acid administration. Analgesic ration was determined with following formula:^52^


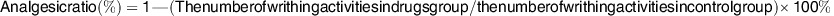


### Statistical analysis

The data collected were analyzed using SPSS 13.0 (Statistical Package for the Social Sciences, Chicago, IL, USA), and the results are expressed as the means±S.D. using one-way analysis of variance and *post hoc* analysis. *P*-values <0.05 were considered statistically significant.

## Abbreviations

MSU: monosodium urate
3D: three-dimension
2D: two-dimension
HUVEC: human umbilical vein endothelial cell
Kin: kinsenoside
Sti: stigmasterol
Ua: ursolic acid
Kae: kaempferol
Gas: gastrodin
CM: conditioned medium
NF-*κ*B: nuclear factor-*κ*B
I*κ*B: inhibitor of NF-*κ*B
IKK: I*κ*B kinase
MAPK: mitogen-activated protein kinase
JNK: c-Jun N-terminal kinase
Erk: extracellular signal-regulated kinase
EC_50_: concentrations for 50% of the maximal effect
COX-1: cyclooxygenase-1
COX-2: cyclooxygenase-2
NSAID: non-steroidal anti-inflammatory drug
Indo: indomethacin
GCC: gradient culture chip
CGC: concentration generator channel
CCC: cell-culturing chamber
PDMS: polydimethylsiloxane
CCK-8: Cell Count Kit-8
BME: cell-basement membrane extract
NALP3: NLR pyrin domain-containing 3
TLR2: toll-like receptor 2
